# Hospital Reform in France

**Published:** 1902-04-12

**Authors:** 


### The Institutional Workshop.

### HOSPITAL REFORM IN FRANCE.

By a Correspondent.

The Paris Municipal Council has recently decided to organise a Permanent Committee of Hygiene to supervise the hospital administration of the Assistance Publique of Paris—that semi-Governmental Department which is more or less equivalent to our poor-law authority. The committee will comprise sanitary engineers, architects, medical men, surgeons, hospital managers, and members of the municipal council. It will have full powers to initiate and carry out reforms. This committee will commence its duties on April 1st next.

These duties will be by no means light, for M. Mourier, the Director of the Assistance Publique, will immediately ask the Municipal Council to sanction the raising of a loan of eighty-three million francs (very nearly £3,300,000), to be expended in completely re-modelling the hospital system.

It is proposed to raze to the ground such famous establishments as the Hôtel-Dieu, the Saint-Louis, Cochin, and Les Enfants-Malades hospitals, and to replace them by erecting institutions of very different outward aspect and internal organisation, in the suburbs.

Most visitors to Paris have been impressed, if not moved to admiration, by the immense solidly-built hospitals, which look as though they were intended to endure for all time. Much may be said for many of these institutions as architectural monuments, but hygienists bring against them a heavy indictment. Briefly, the commission reported that massive buildings of stone, often adorned inside and out with columns and pillars, containing long corridors, huge lofty rooms, were insanitary in the extreme, because:

(1) They afford innumerable harbours for dust.

(2) They are difficult to (*a*) light, (*b*) ventilate, and (*c*) warm.

(3) The bringing together of a great number of patients in huge rooms is (*a*) inconvenient for proper nursing, a danger to (*b*) the patients, and (*c*) the public.

(4) The establishment and upkeep of such institutions in the centres of big cities involves undue expense.

(5) The presence of a great number of patients in hospitals in busy centres of population is inimical to the recovery of invalids and a grave source of danger to public health.

Not content with the comparatively easy task of destructive criticism, the commission put forward a policy of reform.

The hospital of the future is to be something quite different to the old ideal. The grand solidity of stone masonry is to give place to moderate-sized isolated pavilions, built of brick and iron. Instead of long, high-ceilinged rooms to accommodate 100 or more patients, there will be smallish rooms with two or three beds. The rooms will be provided with very little furniture, and all of enamelled iron, while the walls will be washable and nicely painted. The damp sponge is to banish brooms and dusters. Whenever possible the building will consist of a ground floor over a semi-basement, or a ground and first floor only. Different diseases will be relegated to separate pavilions. A central building will be used for administrative purposes, and a more distant one will be fitted up to provide hot water for cooking, baths, and washing, steam for heating purposes (and promoting ventilation), and generating electricity for lighting. Operating theatres are to be contained in three different pavilions, for (1) the ordinary cases; (2) infectious patients; and (3) doubtful cases. Infectious pavilions will be placed as far apart as possible, and provided with separate nursing and other accommodation. All these pavilions will be erected in park-like grounds, on permeable soil, in an elevated position, and at some distance from agglomerations of buildings. An ideal hospital colony shall only provide from 200 to 300 beds; never more than 500.

Given their 83 millions of francs, it will be the task of the Assistance Publique to bring into existence a number of hospital colonies as nearly approaching the above ideal as possible. The task will not be an easy one. Modern hygienists hold that in ordinary towns one, or at most one and a half hospital bed per 1,000 inhabitants will suffice; but that does not apply to great cities. At present Paris possesses one hospital bed for every 208 inhabitants: experience shows that the proportion ought to be one bed for every 75 inhabitants. There is, therefore, much to be done.

Moreover, the new hospitals are to be in sparsely populated suburbs, the pavilions not nearer than 700 yards from any other buildings. On the other hand, the hospital colonies will have to be in neighbourhoods well served by trains or tramways, so that relations and friends of patients may easily visit the invalids.

It should not be forgotten that owing to the Paris hospital system being practically entirely in the hands of the Assistance Publique, the problems of catering, upkeep and laundry work are much simplified. For instance, an admirable central laundry does all the washing under conditions ensuring safety and economy. No doubt the new order of things, when in full working trim, will add somewhat to the labours of the central administration, and the difficulties of adequate supervision will be greater; but such drawbacks are not very alarming.

Of course, it is recognised that in such a large crowded city as Paris, hospitals in the central parts will always be required. But these will be few in number and small, being reserved for accidents or the accommodation of patients unable to bear transport even to the suburbs. It is held, however, that owing to their reduced number and size, even these town establishments may be to a large extent isolated in gardens and fitted up more in accordance with the present day demands of sanitarians.

The reforms about to be inaugurated are undoubtedly of a radical nature, but unquestionably they will meet with the approval of hygienists, and are eminently calculated to foster the confidence of patients and the public generally. The new Committee of Hygiene, with its drastic powers, is largely representative, and the presence of others than medical men and hospital officials will broaden the views of the administration and enlist outside sympathy.

Among other benefits to be expected of such a system is an incalculable saving of waste of money and energy. All who have given any attention to the matter know how often charitable agencies are apt to overlap in certain directions, while leaving other ground quite unprovided for. We know what it is to see some hospitals with their wards dangerously crowded, while others (through lack of patients or funds) have many beds unoccupied. A central authority can adjust matters better, so that more real work can be done at less cost, than where a multiplicity of authorities jostle each other, often clashing in regrettable rivalry.

It is quite possible to preserve a goodly amount of local autonomy, even when these large establishments are under a central control. I know that this is the case with the splendid institutions for women and children, maintained by the Paris Municipality, which I have visited. Each little community lived its own life, under its own guardians, while benefiting by the co-operation and dovetailing which central control made possible.

I have no doubt that the new alliance between the Municipality and the Assistance Publique will lead to equally happy results with the Paris hospitals.

